# Has *Coxiella burnetii* (Q fever) Been Introduced into New Zealand?

**DOI:** 10.3201/eid0901.010305

**Published:** 2003-01

**Authors:** Erik Greenslade, R. Beasley, Lance Jennings, Alistar Woodward, Philip Weinstein

**Affiliations:** * Wellington School of Medicine, Wellington, New Zealand; † Canterbury Health Laboratories, Christchurch, New Zealand

**To the Editor:** New Zealand has been an exception to the panglobal distribution of *Coxiella burnetii* (*1*), the causative organism of Q fever, as shown in a 1990–1991 study (*2*) of 12,556 sheepdogs and 2,181 aborting cattle, all seronegative for *C. burnetii.* In 1997, the *Rabbit hemorrhagic disease virus* (RHDV) was illegally imported from Australia into Central Otago, New Zealand, for the purpose of rabbit control. The unknown source and purity of RHDV, and the potential use of infected rabbits or their organs to transport it, meant that *C. burnetii* could have been coincidentally introduced along with the RHDV-infected rabbit material. To establish whether this occurred, we examined serum specimens from 97 participants enrolled in the RHDV human health study for antibodies to Q fever (*3*).

*C. burnetii* is a very infective organism; it can remain viable for long periods in harsh environmental conditions (*1*). The primary route of human exposure is aerosol dispersion (*1*), and airborne agricultural dusts containing the organism have been implicated in the infection of distant communities (*4*).

Wild rabbits are part of the extensive reservoir of *C. burnetii* in the animal kingdom (*1*) and have been linked with Q fever (*5*). In the United States, 53% of wild rabbits and 39% of wild jackrabbits were found to have antibodies to *C. burnetii,* and the organism has been isolated from both these species (*6*); in Nova Scotia, 49% of hares had antibodies to *C. burnetii* (*7*). In Australia, Q fever is estimated to result in at least 1,700 weeks of lost work time annually, primarily affecting people in the eastern states (*8*).

We could not find evidence of *C. burnetii* in Australian rabbits despite the frequency of Q fever in that country (*8*) and reports of extensive *C. burnetii* infection of rabbits in other countries (*6*). However, the presence of RHDV-infected rabbits in the part of Australia where Q fever is most often reported (*8*,*9*) suggested that the rabbit tissue imported to New Zealand in 1997 might have been infected with *C. burnetii.*

A local lawyer enrolled the study participants anonymously on the basis of their possible exposure to the illegally imported rabbit material. These participants provided serum samples and answered interviewer-administered questionnaires 15 weeks after the first confirmation of the virus (*3*). As a result of the controversy and potential legal proceedings surrounding the circumstances of the biosecurity breach, several questions were considered too sensitive and were not asked; these included the participants’ travel history and exact details of their roles in processing the infected rabbit material. However, in many cases the participants volunteered additional information regarding their exposures.

Nearly all participants (86/97) had had contact with rabbits, and more than half (53/97) appeared to have had contact with RHDV-infected rabbit material. Anecdotal reports suggest that heavy exposures occurred during the harvesting and processing of infected rabbit body organs, spraying of infective organ mixtures onto bait, and distribution of these infective baits by air and ground over 136,000 hectares (340,000 acres).

Thirteen persons were considered to have had a variety of inhalation exposures. Four persons mentioned tasting, smelling, breathing, or having the spray blown in their face for 3–5 hours; another four participants referred to their involvement in spraying. Another four participants had concerns about their inhalation exposures, and one person was included on the basis of exposure while shoveling carrots mixed with infected rabbit blood and molasses. Many additional aerosol exposures likely occurred that the researchers could not specifically identify.

Of the 97 serum samples, 3 were classified as positive to *C. burnetii,* 1 as equivocal, and 93 as negative by using an enzyme-linked immunosorbent assay (PanBio IgG Cat No QFG 100, Brisbane, Australia). On the basis of a single test, determining how many of the results were false positive is difficult: an assay specificity estimate of 95.7% (*10*) suggests we might expect to correctly identify 93 negative serum samples in a population of 97 with no exposure to Q fever.

No evidence of an association between seropositivity and exposure to infective rabbit material was apparent. The most strongly positive result was found in a person who had described no major exposures. One positive result occurred among 39 persons with possible direct contact (i.e., open wound) exposure, and another among 17 persons who had eaten possibly infected rabbit material. None of the 13 persons with inhalation exposures, the 2 persons with needlestick injuries, or the 1 person with a definite bait consumption exposure had serum samples that were positive or equivocal. Likewise, none of the seven persons with reported health problems had serum samples that were either positive or equivocal. The low prevalence of antibodies to *C. burnetii* in the participants in our study (3/97) indicates that most were very unlikely to have had contact with the organism. If the results are true positives, the source of the infection was quite likely outside of New Zealand. However, considering the heavy exposures associated with the cultivation and harvesting of RHDV in live rabbits and the known infectivity of Q fever, *C. burnetii* was not likely to have been introduced inadvertently to New Zealand at the same time as RHDV.
